# Supplementary material for: Comprehensive Analysis of Metabolites in Brews Prepared from Naturally and Technologically Treated Coffee Beans
Source: Antioxidants (Basel). 2022 Dec 30;12(1):95. doi: 10.3390/antiox12010095 (PMC9855040; doi:10.3390/antiox12010095)
Supplement: Supplementary file 1 [file antioxidants-12-00095-s001.zip › Supplementary Figures.pdf]

Comprehensive Analysis of Metabolites in Brews Prepared from Naturally and Technologically Treated Coffee Beans

Magdalena Jeszka-Skowron, Robert Frankowski , Agnieszka Zgoła-Grze´skowiak and Julia Płatkiewicz

Supplementary Figures

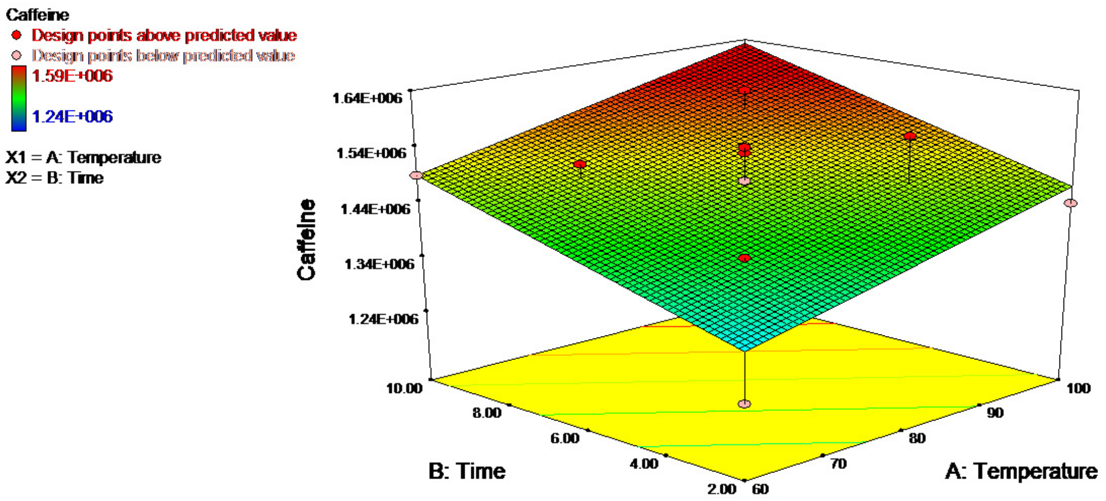

Supplementary Figure S1. Model graph of caffeine for Arabica Mogiana medium roasted coffee brew

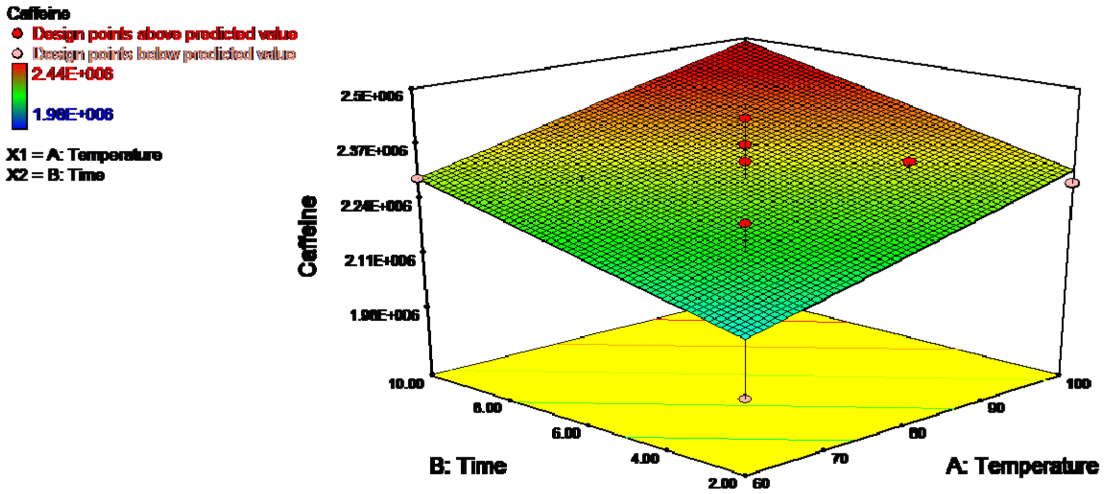

Supplementary Figure S2. Model graph of caffeine for Robusta Vietnam green coffee brew

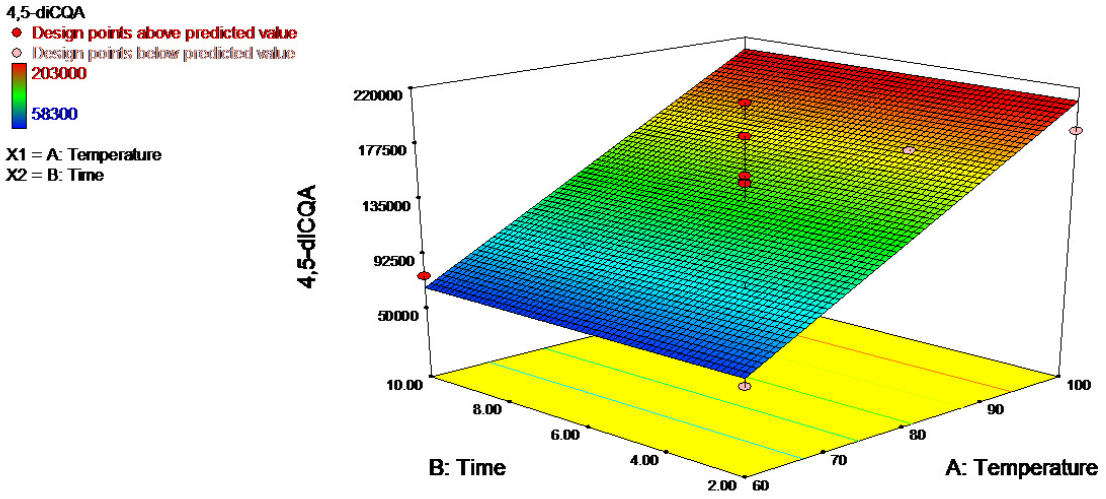

Supplementary Figure S3. Model graph of 4,5-diCQA for Robusta Vietnam green coffee brew

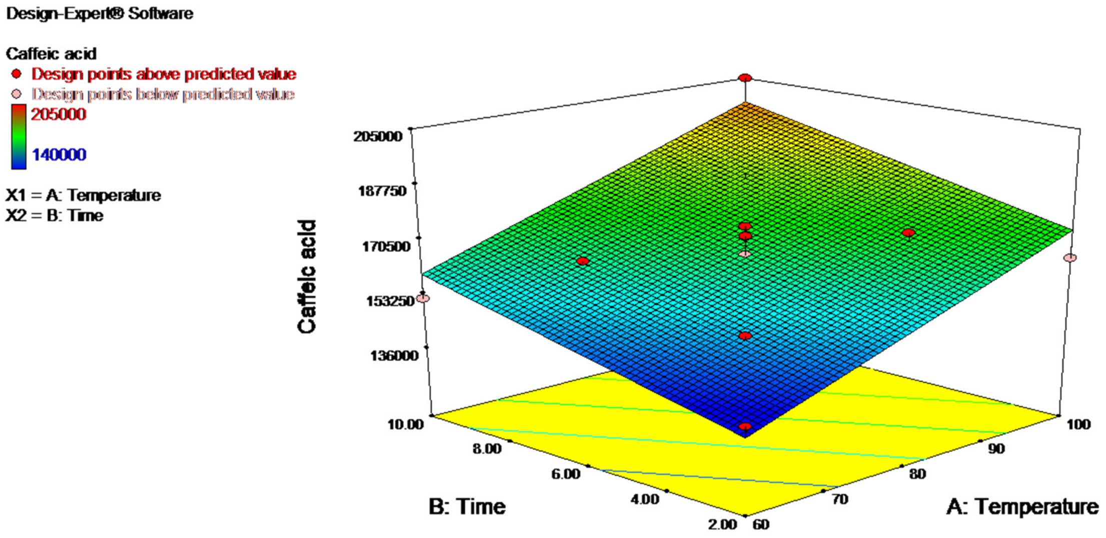

Supplementary Figure S4. Model graph of caffeine for Robusta Vietnam medium roasted coffee brew

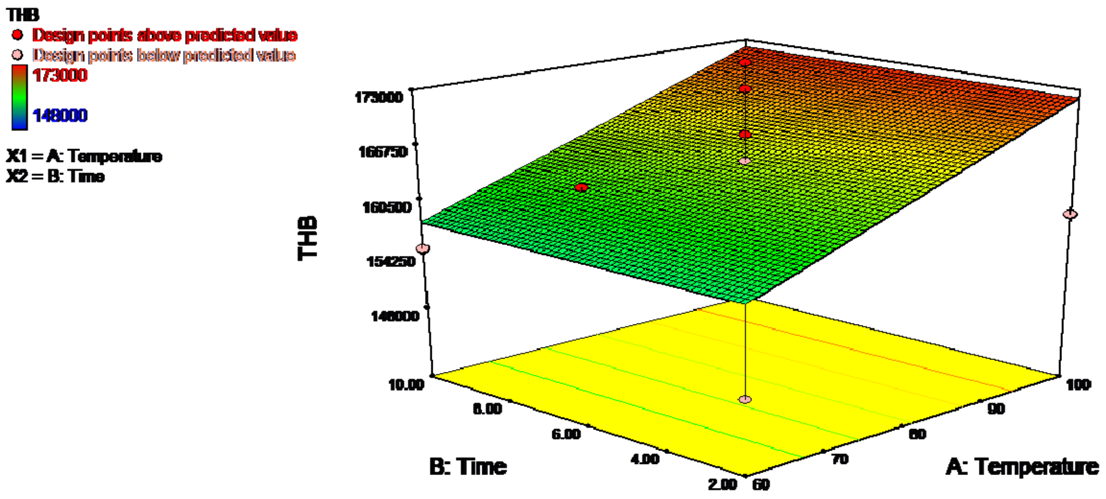

Supplementary Figure S5. Model graph of theobromine (THB) for Robusta Vietnam medium roasted coffee brew
