# Supplementary material for: Comprehensive Analysis of Metabolites in Brews Prepared from Naturally and Technologically Treated Coffee Beans
Source: Antioxidants (Basel). 2022 Dec 30;12(1):95. doi: 10.3390/antiox12010095 (PMC9855040; doi:10.3390/antiox12010095)
Supplement: Supplementary file 1 [file antioxidants-12-00095-s001.zip › Supplem_Tables.pdf]

Comprehensive Analysis of Metabolites in Brews Prepared from Naturally and Technologically Treated Coffee Beans

Magdalena Jeszka-Skowron, Robert Frankowski , Agnieszka Zgoła-Grze´skowiak and Julia Płatkiewicz

Supplementary Tables

Supplementary Table S1. LC mobile phase gradient and MS source parameters

Mobile phase gradient (percentage of acetonitrile)	Parameters of source
0 min 7%,	curtain gas 20 psi
2 min 15%,	nebulizer gas 45 psi
5 min 40%,	auxiliary gas 45 psi
6 min 60%	temperature 450 °C
6.5 min 100%	collision gas medium
8.6 min 100%	ion spray voltage 4500 V
	entrance potential 10 V

Supplementary Table S2. Mass spectrometer parameters applied for the determination of compounds

Compound	DP[a] [V]	Analytical transition	CE[b] [eV]	EP[c] [V]	CXP[d] [V]	Confirmatory transition	CE [eV]	EP [V]	CXP [V]
Trigonelline	50	138 → 94	30	10	4	138 → 110	30	10	5
Theanine	35	175 → 158	17	10	9	175 → 84	30	10	4
Quinic acid	60	193 → 147	13	5	9	193 → 111	21	5	5
Nicotinamide	20	123 → 80	28	10	2	123 → 96	28	10	4
Nicotinic acid	30	124 → 80	30	10	3	124 → 78	32	10	3
Serotonin	50	177 → 160	9	10	12	177 → 132	31	6	7
Theobromine	40	181 → 110	31	10	5	181 → 138	25	10	7
Theophylline	50	181 → 96	33	10	4	181 → 124	27	10	6
5-CQA	50	355 → 163	18	5	10	355 → 145	45	3	7
Caffeine	25	195 →138	26	10	5	195 → 110	32	10	5
4-CQA	50	355 → 163	18	5	10	355 → 145	45	3	7
3-CQA	50	355 → 163	18	5	10	355 → 145	45	3	7
Caffeic acid	48	181 → 163	15	8	9	181 → 135	27	6	7
Melatonin	85	233 → 174	19	5	10	233 → 159	40	6	10
Ferulic acid	45	195 → 177	15	4	10	195 → 145	23	7	7
p-Coumaric acid	45	165 → 147	15	3	7	165 → 119	25	4	6
3,4-diCQA	55	517 → 163	26	5	11	517 → 499	13	5	15
3,5-diCQA	55	517 → 163	26	5	11	517 → 499	13	5	15
4,5-diCQA	55	517 → 163	26	5	11	517 → 499	13	5	15

[a]DP – declustering potential, [b]CE – collision energy, [c]EP – cell entrance potential, [d]CXP – cell exit potential

Supplementary Table S3. LC-MS/MS parameters for analyzed compounds (RT – retention time; LOD – limit of detection; LOQ – limit of quantification)

Compound	RT [min]	Intercept	Slope	R^2	Range [μg mL^{-1}]		LOD [μg mL^{-1}]	LOQ [μg/mL^{-1}]
					min	max		
Trigonelline	0.73	66728	42979115	0.9985	0.001	0.05	0.00002	0.00006
Theanine	0.76	22629	21128693	0.9994	0.0005	0.2	0.00004	0.00014
Quinic acid	0.77	2123	345922	0.9992	0.0025	1.0	0.00465	0.01551
Nicotinamide	0.91	36020	39248609	0.9996	0.0005	0.05	0.00001	0.00005
Nicotinic acid	0.92	7691	22552053	0.9998	0.0005	0.05	0.00006	0.00021
Serotonin	0.97	14241	22007112	0.9993	0.0005	0.1	0.00003	0.00008
Theobromine	1.42	-61.64	4336884	1.0000	0.0005	0.5	0.00009	0.0003
Theophylline	2.02	10859	6508748	0.9991	0.0005	0.2	0.00012	0.0004
5-CQA	2.53	1786	781478	1.0000	0.0025	2.5	0.00047	0.00156
Caffeine	2.61	43041	18229109	0.9992	0.001	0.2	0.00004	0.00012
4-CQA	3.42	4750	551201	0.9996	0.0025	2.5	0.00072	0.00239
3-CQA	3.70	6861	3902774	0.9999	0.002	1.0	0.0001	0.00034
Caffeic acid	4.36	8294	7322094	0.9998	0.0005	0.5	0.0007	0.00233
Melatonin	5.02	48058	135783791	0.9992	0.00025	0.025	0.00000106	0.00000353
Ferulic acid	5.1	25627	12881257	0.9998	0.0005	0.5	0.00044	0.00148
p-Coumaric acid	5.38	14546	7028294	0.9997	0.0005	0.5	0.00043	0.00144
3,4-diCQA	5.32	5745	221049	0.9993	0.005	2.5	0.00125	0.00416
3,5-diCQA	5.53	-3227	246328	0.9998	0.005	2.5	0.00149	0.00497
4,5-diCQA	5.69	709	270530	0.9995	0.005	0.5	0.00102	0.00341

Supplementary Table S4. ANOVA for Response Surface Models Arabica Brazil Mogiana green coffee

Coefficient Removed	t for H0 Estimate	Coeff=0	Prob > lt l	R-Squared	MSE
B	2090.00	0.81	0.4392	0.1271	3.258E+007
A	3230.00	1.27	0.2319	0.0000	3.421E+007

Std. Dev.	5849.22	R-Squared	0.0000
Mean	97500.00	Adj R-Squared	0.0000
C.V. %	6.00	Pred R-Squared	-0.1736
PRESS	4.818E+008	Adeq Precision	

ANOVA for Response Surface Mean Model – nicotinic acid

Analysis of variance table [Partial sum of squares - Type III]

Source	Sum of Squares	df	Mean Square	F Value	p-value Prob > F	
Model	0.000	0				
Residual	4.106E+008	12	3.421E+007			
Lack of Fit	2.933E+008	8	3.666E+007	1.25	0.4419	not significant
Pure Error	1.173E+008	4	2.931E+007			
Cor Total	4.106E+008	12				

Removed	Coefficient Estimate	t for H0 Coeff=0	Prob > lt l	R-Squared	MSE
B	92000	0,198752	0,8464	0,196802	9,78E+11
A	726000	1,641724	0,1289	1,89E-15	1,12E+12

Std. Dev.	1056369,569	R-Squared	0
Mean	5690000	Adj R-Squared	0
C.V. %	18,56537028	Pred R-Squared	-0,17361
PRESS	1,57158E+13	Adeq Precision	

ANOVA for Response Surface Mean Model - 4-CQA

Analysis of variance table [Partial sum of squares - Type III]

Source	Sum of Squares	df	Mean Square	F Value	p-value Prob > F	
Model	0	0				
Residual	1,3391E+13	12	1,11592E+12			
Lack of Fit	6,62E+12	8	8,275E+11	0.48885	0.8195	not significant
Pure Error	6,771E+12	4	1,69275E+12			
Cor Total	1,3391E+13	12				

ANOVA for Response Surface Mean Model – 3-CQA

Analysis of variance table [Partial sum of squares - Type III]

Source	Sum of Squares	df	Mean Square	F Value	p-value Prob > F	
Model	0.000	0				
Residual	5.524E+013	12	4.603E+012			
Lack of Fit	4.725E+013	8	5.906E+012	2.96	0.1549	not significant
Pure Error	7.988E+012	4	1.997E+012			
Cor Total	5.524E+013	12				

Std. Dev.	2.146E+006	R-Squared	0.0000
Mean	4.530E+007	Adj R-Squared	0.0000
C.V. %	4.74	Pred R-Squared	-0.1736
PRESS	6.483E+013	Adeq Precision	

Supplementary Table S5. ANOVA for Response Surface Models Arabica Brazil Mogiana roasted coffee

ANOVA for Response Surface Linear Model - caffeine

Analysis of variance table [Partial sum of squares - Type III]

Source	Sum of Squares	df	Mean Square	F Value	p-value Prob > F	
Model	58010000000	2	29005000000	8,2677	0.0076	significant
A-Temperature	25205000000	1	25205000000	7,184533	0.0231	
B-Time	32805000000	1	32805000000	9,350867	0.0121	
Residual	35082307692	10	3508230769			
Lack of Fit	29362307692	6	4893717949	3,42218	0.1269	not significant
Pure Error	5720000000	4	1430000000			
Cor Total	93092307692	12				

Std. Dev.	59230,31968	R-Squared	0,623145
Mean	1480769,231	Adj R-Squared	0,547774
C.V. %	3,999969641	Pred R-Squared	0,0123
PRESS	91947306733	Adeq Precision	10,68416

ANOVA for Response Surface Mean Model – ferulic acid

Analysis of variance table [Partial sum of squares - Type III]

Source	Sum of Squares	df	Mean Square	F Value	p-value Prob > F	
Model	0	0				
Residual	100014123,1	12	8334510,256			
Lack of Fit	50671843,08	8	6333980,385	0,513473	0.8041	not significant
Pure Error	49342280	4	12335570			
Cor Total	100014123,1	12				

Std. Dev.	2886,955188	R-Squared	0
Mean	6255,384615	Adj R-Squared	0
C.V. %	46,15152169	Pred R-Squared	-0,17361
PRESS	117377686,1	Adeq Precision	

Supplementary Table S6. ANOVA for Response Surface Model Robusta Vietnam green coffee

ANOVA for Response Surface Linear Model – Nicotinic acid

Analysis of variance table [Partial sum of squares - Type III]

Source	Sum of Squares	df	Mean Square	F Value	p-value Prob > F	
Model	12112160	2	6056080	5,483871	0.0247	significant
A-Temperature	4960080	1	4960080	4,491427	0.0601	
B-Time	7152080	1	7152080	6,476315	0.0291	
Residual	11043440	10	1104344			
Lack of Fit	8583440	6	1430573,333	2,326136	0.2167	not significant
Pure Error	2460000	4	615000			
Cor Total	23155600	12				

Std. Dev.	1050,877728	R-Squared	0,523077
Mean	12990	Adj R-Squared	0,427692
C.V. %	8,089897832	Pred R-Squared	0,223451
PRESS	17981458,77	Adeq Precision	8,684192

ANOVA for Response Surface Linear Model - caffeine

Analysis of variance table [Partial sum of squares - Type III]

Source	Sum of Squares	df	Mean Square	F Value	p-value Prob > F	
Model	90500000000	2	45250000000	7,084789	0.0121	significant

Std. Dev.	79918,22744	R-Squared	0,586257
Mean	2301538,462	Adj R-Squared	0,503508
C.V. %	3,472382877	Pred R-Squared	0,147749
PRESS	1,31561E+11	Adeq Precision	9,898031

A-Temperature	50000000000	1	50000000000	7,828496	0.0189	
B-Time	40500000000	1	40500000000	6,341082	0.0305	
Residual	63869230769	10	6386923077			
Lack of Fit	49949230769	6	8324871795	2,392205	0.2090	not significant
Pure Error	13920000000	4	3480000000			
Cor Total	1,54369E+11	12				

Coefficient	t for H0				
Removed	Estimate	Coeff=0	Prob > |t|	R-Squared	MSE
B-Time	4070.00	0.39	0.7051	0.8238	5.040E+008

Std. Dev.	22451.04	R-Squared	0.8238
Mean	1.384E+005	Adj R-Squared	0.8078
B-Time	C.V. %	16.22	Pred R-Squared
PRESS	7.314E+009	Adeq Precision	16.355

ANOVA for Response Surface Reduced Linear Model - 4,5-CQA

Analysis of variance table [Partial sum of squares - Type III]

Source	Sum of Squares	df	Mean Square	F Value	p-value Prob > F	
Model	2.593E+010	1	2.593E+010	51.44	< 0.0001	significant
A-Temperature	2.593E+010	1	2.593E+010	51.44	< 0.0001	
Residual	5.545E+009	11	5.040E+008			

Lack of Fit	3.867E+009	7	5.525E+008	1.32	0.4165	not significant
Pure Error	1.677E+009	4	4.193E+008			
Cor Total	3.147E+010	12				

Supplementary Table S7. ANOVA for Response Surface Models Robusta Vietnam roasted coffee

ANOVA for Response Surface Mean Model - 5-CQA

Analysis of variance table [Partial sum of squares - Type III]

Source	Sum of Squares	df	Mean Square	F Value	p-value Prob > F	
Model	0.000	0				
Residual	5.394E+009	12	4.495E+008			
Lack of Fit	4.725E+009	8	5.906E+008	3.53	0.1192	not significant
Pure Error	6.692E+008	4	1.673E+008			
Cor Total	5.394E+009	12				

Std. Dev.	21202.02	R-Squared	0.0000
Mean	4.858E+005	Adj R-Squared	0.0000
C.V. %	4.36	Pred R-Squared	-0.1736
PRESS	6.331E+009	Adeq Precision	

Removed	Coefficient Estimate	t for H0 Coeff=0	Prob > |t|	R-Squared	MSE
B-Time	4400	1,534447	0,1559	0,325398	46174825

Std. Dev.	6795,206044	R-Squared	0,325398
Mean	165076,9231	Adj R-Squared	0,264071
C.V. %	4,116387631	Pred R-Squared	-0,0773
PRESS	811126009,2	Adeq Precision	5,252694

ANOVA for Response Surface Reduced Linear Model - theobromine

Analysis of variance table [Partial sum of squares - Type III]

Source	Sum of Squares	df	Mean Square	F Value	p-value Prob > F	
Model	245000000	1	245000000	5,305922	0.0418	significant

A-Temperature	245000000	1	245000000	5,305922	0.0418	
Residual	507923076,9	11	46174825,17			
Lack of Fit	452723076,9	7	64674725,27	4,686574	0.0772	not significant
Pure Error	55200000	4	13800000			
Cor Total	752923076,9	12				

ANOVA for Response Surface Linear Model - Caffeic acid

Analysis of variance table [Partial sum of squares - Type III]

Source	Sum of Squares	df	Mean Square	F Value	p-value Prob > F	
Model	2402600000	2	1201300000	21,28745	0.0002	significant
A-Temperature	1729800000	1	1729800000	30,65265	0.0002	
B-Time	672800000	1	672800000	11,92225	0.0062	
Residual	564323076,9	10	56432307,69			
Lack of Fit	305523076,9	6	50920512,82	0,787025	0.6230	not significant
Pure Error	258800000	4	64700000			
Cor Total	2966923077	12				

Std. Dev.	7512,144014	R-Squared	0,809795
Mean	166923,0769	Adj R-Squared	0,771754
C.V. %	4,500362774	Pred R-Squared	0,589403
PRESS	1218209388	Adeq Precision	16,73725

ANOVA for Response Surface Mean Model - Quinic acid

Analysis of variance table [Partial sum of squares - Type III]

	Sum of		Mean	F	p-value	

Std. Dev.	20106.13	R-Squared	0.0000
Mean	4.264E+005	Adj R-Squared	0.0000
C.V. %	4.72	Pred R-Squared	-0.1736

Source	Squares	df	Square	Value	Prob > F	
Model	0.000	0				
Residual	4.851E+009	12	4.043E+008			
Lack of Fit	3.798E+009	8	4.747E+008	1.80	0.2981	not significant
Pure Error	1.053E+009	4	2.633E+008			
Cor Total	4.851E+009	12				

PRESS	5.693E+009	Adeq Precision	
Coefficient	Standard	95% CI	95% CI
Factor	Estimate	df	Error
Intercept	4.264E+005	1	5576.44
